# Advancing Preoperative Strategies for Thyroidectomy in Graves’ Disease: A Narrative Review

**DOI:** 10.7759/cureus.48156

**Published:** 2023-11-02

**Authors:** David Elkhoury, Pruthvi Patel, Saalini Sastry, Alireza Tajik, Christina Elkhoury, Michael Montalbano

**Affiliations:** 1 Anatomical Sciences, St. George's University School of Medicine, Saint George's, GRD; 2 Health Sciences, Trident University International, Chandler, USA; 3 Molecular Pharmacology and Toxicology, University of Southern California, Los Angeles, USA

**Keywords:** goiter, graves´disease, thyroid crisis, thyrotoxicosis, antithyroid agents

## Abstract

Graves' disease is an autoimmune disorder characterized by thyroid-stimulating antibodies that can potentially lead to thyrotoxicosis, goiter, skin disease, and eye disease. Available treatment options for Graves' disease include management with antithyroid drugs (ATDs), thyroid ablation with radioactive iodine (RAI), and surgical thyroid gland removal. For individuals unable to reach a normal thyroid hormone level, promptly considering a thyroidectomy is essential. Preoperative strategies to achieve a euthyroid state prevent thyroid storms and minimize postoperative complications and are therefore crucial. While variations in professional guidance exist, this review focuses on standard medical interventions as well as compares respective guidelines set forth by the American Thyroid Association, the European Thyroid Association, the American Association of Clinical Endocrinology, and the American Association of Endocrine Surgeons. There is consensus among these organizations underscoring the importance of rendering patients euthyroid prior to surgery and the use of ATDs. Most guidelines recommend screening for vitamin D deficiency as well as endorse thyroidectomy as the preferred treatment option for hyperthyroidism with skilled surgeons. Nevertheless, discrepancies do become apparent in aspects such as potassium iodide (SSKI) course duration and preoperative dexamethasone administration. By understanding these differing approaches, healthcare professionals can more effectively manage Graves' disease prior to surgery, resulting in improved patient outcomes and enhanced surgical success.

## Introduction and background

Graves' disease is a complex autoimmune disorder that affects the thyroid gland through thyroid-stimulating antibodies (TSAb), which bind to and activate the thyrotropin or thyroid-stimulating hormone (TSH) receptor on the surface of thyroid follicular cells [1]. Consequently, excessive production of thyroid hormones can lead to various conditions, such as thyroid gland enlargement known as goiter and a skin condition called pretibial myxedema. In addition, Graves' disease often involves ophthalmopathy, a unique feature not seen in other causes of hyperthyroidism that makes tissues around the eyes, such as the extraocular muscles and orbital fat, particularly susceptible to the effects of the autoimmune response in Graves' disease. This occurs because TSAbs trigger an autoimmune response, which leads to inflammation and edema. The epidemiological impact of Graves' disease differs substantially by sex, affecting approximately 3% of women and 0.5% of men [2].

Available treatment options for Graves' disease include antithyroid drugs (ATDs), thyroid ablation with radioactive iodine (RAI), and surgical thyroid gland removal. Graves' disease treatment typically begins with the administration of an ATD. For patients who have allergic reactions to ATDs, the American Thyroid Association (ATA) guidelines recommend that the patient receive adequate preoperative treatment with beta-blockers and potassium iodide (SSKI) [3]. However, the European Thyroid Association (ETA) approach involves preoperative vocal cord evaluation and recommends a different list of preoperative drugs such as beta-blockers and glucocorticoids [4]. Moreover, the 2016 ATA guidelines recommend lowering thyroid hormone levels with methimazole and a saturated solution of SSKI before thyroidectomy.

**Figure 1:**
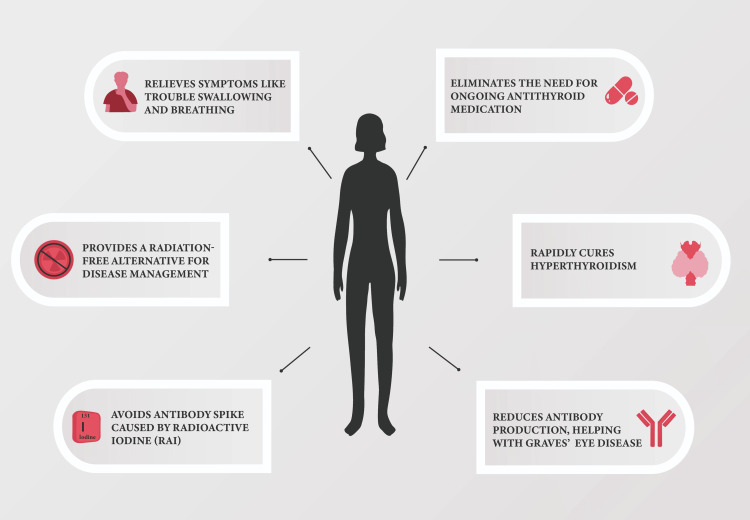
Benefits of Thyroidectomy for Graves' Disease Image Source: With permission from image owner David Elkhoury. Content adapted from 'Surgical Management of Graves’ Disease' by the American Thyroid Association [7].

If patients cannot achieve a normal thyroid hormone level, the ATA guidelines strongly emphasize the importance of conducting an immediate thyroidectomy. The many advantages of thyroidectomy in managing Graves' disease, as emphasized by the ATA, are exemplified in Figure 1. In the context of Graves' disease, patients are more likely to experience surgical complications, particularly vocal cord paralysis and hypocalcemia [5]. As a result, it is recommended that Graves' disease patients should be referred to a facility with a large volume of high-caliber surgical procedures so that the patient population can be carefully managed prior to surgery [6].

Preoperative preparation measures are critical for Graves' disease patients undergoing thyroidectomy. The primary aim of this review is to provide an overview of preoperative treatments and recommendations for optimizing patient care during the preoperative phase in order to achieve favorable surgical outcomes in Graves' disease patients undergoing thyroidectomy. These findings can reinforce preferred healthcare standards and lead to better surgical outcomes for this patient population.

Clinical diagnosis and assessment

Graves' disease often has a distinct clinical presentation, including ophthalmological manifestations and goiter. Assessing the severity of these symptoms can be a reliable diagnostic measure when used in conjunction with laboratory findings [8]. Various diagnostic methods are available to measure thyroid function, including directly analyzing the thyroid hormones (TSH, T3, and T4). However, the total measurements of T3 and T4 can be influenced by binding proteins' availability, rendering them insufficient evidence of metabolic health. For a more accurate assessment, free T3 and T4 measurements are preferred indicators of metabolic status and changes in thyroid hormone synthesis.

Hormone levels (free and bound) can be measured to assess the regulatory process of the hypothalamic-pituitary-thyroid (HPT) axis. Thyrotropin levels serve as one of the most reliable indicators of thyroid disorders. The development of higher-quality thyrotropin assays has resulted in thyrotropin levels becoming a more accurate indicator of HPT axis function than a traditional thyroid function panel. One thyrotropin test may now be used for most patients, dramatically changing screening and early assessments. Additionally, radiolabeled iodine (iodine-131) can be given to study how effectively the thyroid gland absorbs and utilizes iodine over time [9].

Medical management options for Graves’ disease

ATDs and RAI are available to control excessive thyroid hormone production and manage symptoms of Graves' disease. If left untreated, Graves' disease can result in an overproduction of thyroid hormones, likely culminating in a thyroid crisis. People with this condition are particularly susceptible to a thyrotoxic storm, which has a rapid onset and a life-threatening intensification of hyperthyroidism. Patients can present with a high fever, dehydration, marked tachycardia or tachyarrhythmias, heart failure, hepatomegaly, respiratory distress, abdominal pain, delirium, and seizures. [10]. These findings illustrate the importance and complexity of quickly managing thyroid hormone levels.

Antithyroid Medications

Antithyroid medication therapy is the most common first-line treatment in Europe, Asia, and, to a lesser extent, the United States [11]. In addition to the advantages of low cost and safety of administration, continuous antithyroid medication treatment for 12-18 months can improve disease remission compared to no intervention [11,12]. Additionally, maintaining euthyroidism prior to surgery is associated with improved surgical outcomes and postoperative recuperation [13].

Propylthiouracil (PTU), methimazole, and its prodrug carbimazole are the three significant thionamides used to treat Graves' disease and hyperthyroidism. Thionamides block thyroid peroxidase, leading to the inhibition of iodine oxidation, iodination, and coupling of monoiodotyrosine (MIT) and diiodotyrosine (DIT) resulting in reduced synthesis of T3 and T4 hormones [14]. Methimazole is more commonly preferred due to its superior ability to reduce elevated thyroid hormones and decrease the risk of liver damage [15]. On the other hand, PTU is utilized in specific clinical scenarios. PTU is vital in potentially fatal emergencies, such as thyroid storms, and for pregnant women during the earliest stage of gestation to avoid the associated risks of methimazole-related congenital disabilities. The preference for PTU in such cases is due to its distinctive inhibition of the D1 isoform of the deiodinase enzyme, resulting in reduced synthesis of T3 and T4 hormones, with the former capable of being rapidly reduced as much as 50% [16]. Moreover, PTU provides an option for patients who develop adverse reactions to methimazole and are not seeking surgery or radioiodine treatment [17]. However, the FDA has provided a cautionary stance against the use of PTU due to the potential for severe liver injury and acute liver failure.

The use of ATDs typically takes six weeks to achieve a euthyroid state. When achieving euthyroidism with traditional treatment is challenging, considering adjuvant therapies to prepare patients for surgery is crucial [18]. Several studies have examined the effectiveness of incorporating cholestyramine as a treatment option for patients with Graves' disease. Notably, in 2016, Chae et al. utilized cholestyramine as an adjunct therapy to the standard treatment plan for individuals with treatment-resistant Graves' disease. This study suggests that cholestyramine can produce preoperative euthyroidism quickly and easily [19]. Although refractory cases with antithyroid medications are uncommon, isolated cases have demonstrated that adjunctive therapies can quickly restore a normal thyroid state [20].

Beta-Blockers

Many of the abnormalities in Graves’ disease patients, including tachycardia, anxiety, palpitations, and increased respiration, correspond to those caused by external stimulation of beta-adrenergic receptors. Therefore, beta-blockers have been utilized in combination with antithyroid medications to resolve these symptoms before surgery. Nonselective beta-blockers, such as propranolol, may reduce sympathetic function by blocking beta receptors but also have direct antithyroid effects by reducing T4 conversion to T3 by interfering with the activity of 5’-deiodinase. Although beta-blockers are a first-line treatment, patients should be closely monitored for risk of heart failure. Patients with prior heart failure or thyroid storm heart failure may benefit more from esmolol due to its shorter half-life, cardioselectivity, and fewer inotropic side effects. In contrast, those with stable asthma or mild obstructive airway disease may benefit most from atenolol or metoprolol [21].

Alternative Therapies

Before surgery, beta-blockers and SSKI should be given to patients who urgently need a thyroidectomy or are allergic to ATDs. SSKIs rapidly decrease thyroid hormone secretion by inhibiting iodine uptake into thyroid cells. As a result, they work quickly and can show impact within a week. Thionamide, on the other hand, blocks thyroid hormone production and has a slow onset of action, taking about three to four weeks to show the full effect. ATDs are commonly favored as the treatment of choice for patients likely to have remission, such as women, individuals with mild disease, small tumors, and suboptimal or low immune responses. If symptoms persist for over six months, strong consideration should be given to pursue a thyroidectomy or RAI [17].

RAI is a treatment for hyperthyroidism, particularly in Graves' disease. This involves administration of RAI (iodine-131) orally or intravenously. As the overactive thyroid gland takes up RAI, the resulting radiation damages the overactive thyroid tissue, thereby decreasing thyroid hormone production and possible recurrence of Graves' disease [22]. While often successful in achieving remission, Schneider et al. found that nearly 23% of patients failed RAI, requiring a second dose or surgery for sustained treatment [23]. Sundaresh et al. also reported a radiation thyroiditis incidence of 1.2% among individuals who underwent RAI treatment, which worsens hyperthyroid symptoms and overall patient health [24]. However, because it is a non-invasive approach, RAI is a viable option for high-risk surgical patients such as the elderly or those with previous neck surgeries. RAI is also recommended for patients who have failed ATD therapy because it provides a permanent, minimally invasive alternative.

Comparing indications for surgery

While ATDs and RAI have been the most common treatments in the United States, surgery may be recommended for patients with Grave’s disease who are nonresponsive to ATDs and have relapsed. According to most medical association guidelines, surgery can be an option for the indications such as: (1) patients with large goiters (≥80g); (2) patients with goiters that lead to airway obstruction and dysphasia; (3) patients with moderate to severe ophthalmopathy; (4) patients who are breastfeeding or planning on becoming pregnant in <6 months; (5) patients who have primary hyperthyroidism after RAI; (6) suspected thyroid malignancy. Patients with toxic multinodular goiter or toxic adenoma with obstructive symptoms, concurrent hyperparathyroidism, and absolute hyperthyroidism are also noted [25].

Although ATDs and RAIs are the first lines of treatment, a recent study by Cipolla et al. claims that a total thyroidectomy may be a viable long-term alternative for low-income patients as it is a cost-effective option and the risk of recurrent hyperthyroidism is very low [26]. They further evaluated 594 cases for the predominant factors, indicating a total thyroidectomy as the best option, and found that the patients who were recommended for surgery exhibited more than one indication [26].

## Review

Preoperative management

The guidelines of the ATA, the ETA, the American Association of Clinical Endocrinologists (AACE), and the American Association of Endocrine Surgeons (AAES) to improve outcomes in patients considering thyroidectomy have been summarized in Table 1.

**Table 1:** Comparative Assessment of Thyroidectomy Guidelines of ATA, ETA, AAES, and AACE AACE: American Association of Clinical Endocrinologists; AAES: American Association of Endocrine Surgeons; ATA: American Thyroid Association; ATD: antithyroid drug; BB: beta-blockade; ETA: European Thyroid Association; GCs: glucocorticoids; MMI: methimazole; NS: not specified; SSKI: saturated solution of potassium iodide

Guideline Aspect	ATA	ETA	AAES	AACE
	References: [4, 7, 17, 27]	References: [28-30]	References: [31,32]	References: [3,33]
Voice Assessment	NS	NS	Recommended for diagnosis of thyroid disease	NS
Vocal Cord Evaluation	Recommended for anaplastic thyroid cancer patients	Preoperative assessment recommended	NS	Preoperative assessment recommended
Preoperative Medication	ATD (MMI) ± β-blockade	ATD (MMI), β-blockers, GCs	ATD (MMI)	MMI
Alternate Medications	SSKI, β-blockade, GCs, or cholestyramine in urgent cases or allergic reactions to ATD	SSKI (if needed), for urgent thyroidectomy before adequate control of hyperthyroidism is achieved	NS	In cases of allergy or urgency, give BB + SSKI
Medication Duration	Give SSKI for 10 days prior to surgery	Give SSKI for 10 days prior to surgery	Give SSKI for 7-10 days prior to surgery	Give SSKI or inorganic iodine for 10 days prior to surgery
Preoperative Assessment and Correction of Vitamin D Deficiency	Yes	Yes	Yes	NS
Preoperative Dexamethasone	NS	NS	Consider a single preoperative dose of dexamethasone to reduce nausea, vomiting, and pain	NS

Each organization agrees on the importance of clinical assessment for personal and family history of thyroid disease as well as rendering patients euthyroid preoperatively using antithyroid medications such as methimazole, β-adrenergic blockers, and glucocorticoids [27,29,31]. Additionally, most guidelines recommend screening for vitamin D deficiency prior to surgery [3,29,31]. The guidelines unanimously support thyroidectomy as the preferred procedure for hyperthyroidism, emphasizing the need for a skilled, high-volume surgeon to ensure better surgical outcomes [3,29,31,33].

However, observable differences are evident in a few specified areas. Specifically, the ATA, ETA, and AACE offer a shared recommendation for a 10-day course of administering SSKI before surgery [3,17,29]. In contrast, the AAES advocates a 7- to 10-day course of administering SSKI before surgery [31]. Choosing between a 10-day course versus a 7- to 10-day course requires weighing the potential advantages and disadvantages of treatment duration. Prolonged administration may extend thyroid control but also increase the risk of unwanted side effects such as hives, joint pain, swelling of extremities and facial areas, and lymph gland enlargement. To our knowledge, no studies have directly compared a 10-day course versus a 7- to 10-day course of SSKI in patients with Graves’ disease undergoing thyroidectomy.

Additionally, the AAES introduces the proposal of preoperative administration of dexamethasone to ameliorate potential discomfort that may encompass symptoms such as nausea, vomiting, and pain. The ATA, ETA, and AACE do not include such a provision. Understanding this distinction can be important for physicians in order to comfort patients, manage symptoms, and mitigate postoperative discomfort. In one study, the administration of a single dose of 8 mg of intravenous dexamethasone with general anesthesia induction significantly reduced both postoperative nausea at 24 hours and vomiting for up to 72 hours, reducing the need for additional antiemetic medication [34]. Similarly, Worni et al. reported similar outcomes, finding that a preoperative administration of a single dose significantly diminished postoperative nausea, vomiting, and pain while enhancing vocal function within 48 hours following thyroidectomy [35].

Differences in surgical indications can be seen in a comparison of the guidelines set forth by the ATA with the ETA, the AAES, and the AACE. The ETA supports ATA guidelines and recommends surgery for patients with large goiters, primary hyperparathyroidism, and patients who are not responsive or wish to avoid ATD and RAI [29]. The AAES adds that many asymptomatic goiters may not warrant surgery, but many surgeons consider surgery for goiters that cause dyspnea, dysphagia, tracheal or esophageal compression, or thoracic outlet syndrome. They also emphasize that patients with hyperthyroidism should be recommended for surgery when RAI is contraindicated or produces unwanted results in the patient [36]. The AACE aligns with the ATA guideline and emphasizes that surgery should be carefully considered in patients with specific comorbidities, including cardiopulmonary disease and terminal cancers [3].

Surgical techniques

**Figure 2:**
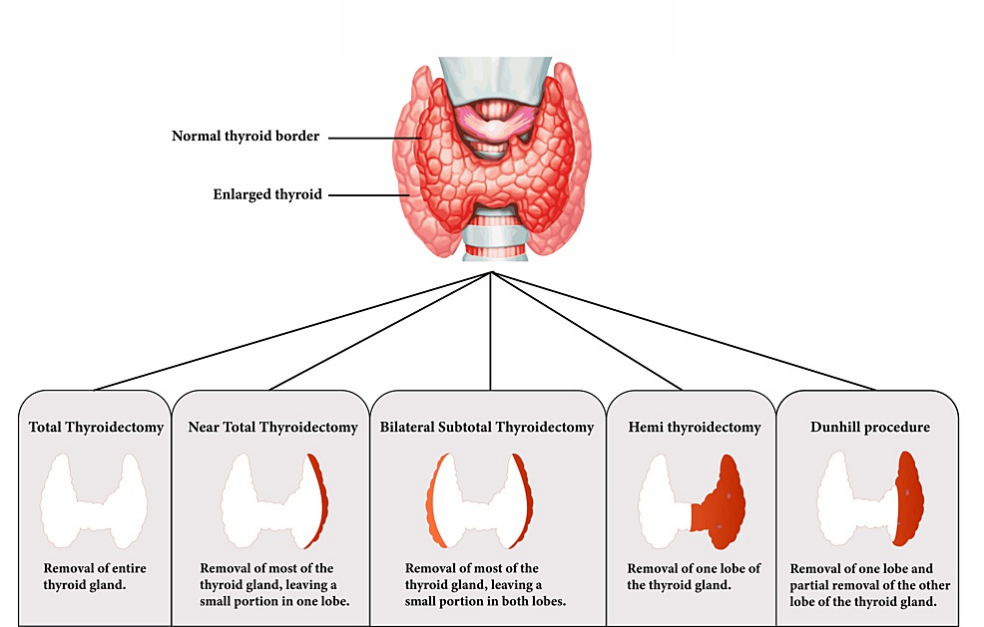
A Comparative Overview of Thyroidectomy Procedures for Treatment of Graves’ Disease Image Source: With permission from image owner David Elkhoury

Multiple surgical approaches are available to treat Graves' disease, including total thyroidectomy, near-total thyroidectomy, bilateral subtotal thyroidectomy, hemi thyroidectomy, and the Dunhill method (Figure 2). While the choice to undergo surgery for Graves' disease is a difficult one that requires careful consideration and discussion, the ATA, ETA, and AAES guidelines suggest that total or near-total thyroidectomy is superior to subtotal thyroidectomy [17,29,36]﻿. The strategic excision of a substantial portion of the thyroid gland presents a benefit in minimizing the risk of Graves' disease relapse and reducing the potential recurrence of symptoms.

Postoperative considerations

Surgery to remove thyroid tissue in patients with hyperthyroidism has been linked to increased complication rates. One specific concern is that patients diagnosed with Graves' disease can experience a thyrotoxic crisis, also known as a thyroid storm. To mitigate the risk of a thyroid storm, a course of Lugol's iodine is commonly given prior to surgical intervention to promptly reduce hormone production within hours of administration [2]. This preoperative administration of Lugol's iodine aims to prevent the sudden surge of thyroid hormones, minimizing the chances of complications for patients undergoing treatment.

Postoperative transient hypocalcemia is also a common consequence following thyroid surgery. Clinical manifestations of hypocalcemia may present in patients as unwanted symptoms, such as tingling and muscle spasms. Clinical studies have demonstrated that oral calcium and vitamin D supplements before total thyroidectomy surgery can significantly decrease the occurrence and symptoms of hypocalcemia [32,37]. Identifying the parathyroid thyroid glands during thyroid surgery is crucial for preventing damage to the glands that could cause short-term or permanent hypocalcemia in patients [38]. Methylene blue spray may also be an effective and safe method for improving thyroid surgery outcomes since a recent study of patients undergoing thyroid surgery, using a methylene blue spray helped in 82% of cases to visualize and preserve parathyroid glands [39].

Future directions

Despite the extensive literature and existing guidelines available to healthcare professionals, there is no consensus on magnesium among the guidelines reviewed here. Nevertheless, there is a growing body of research focused on examining the complex interactions between magnesium imbalances and their possible implications for hypocalcemia in the short- and long-term after thyroidectomy. Nellis et al. found that the odds of developing hypocalcemia were the greatest for patients with magnesium metabolism disorders [40]. In addition, Liu et al. showed that magnesium supplements before, during, and after thyroid surgery could be an effective prophylaxis against surgical hypocalcemia [41].

Although open thyroidectomy is known for safety and efficacy, there is a growing interest in developing minimally invasive techniques that aim to reduce surgical trauma, shorten hospital stays, and increase postoperative recovery. These innovative techniques also aim to improve patient outcomes and overcome the challenges of working at a restricted surgical site during thyroid surgeries. In light of these challenges, researchers have conducted comparative studies to examine the surgically-related complications between robotic-assisted thyroidectomy and conventional open thyroidectomy. Thus far, the findings suggest that robotic-assisted thyroidectomies are associated with longer operating times, extended hospital stays, and a higher incidence of temporary recurrent laryngeal nerve injury [42]. With the quick advancement of endoscopic methods and tools, thyroidectomies can also be carried out with no visible scars on the neck specifically. In particular, the transoral endoscopic thyroidectomy vestibular approach (TOETVA) has recently emerged as a potentially viable and safe treatment option with notable clinical and aesthetic results, particularly for patients with Graves' disease requiring thyroid surgery [43].

## Conclusions

This literature review emphasizes the significance of preoperative strategies for thyroidectomy in Graves' disease patients. Achieving euthyroid status before surgery has been linked to better surgical results and a faster postoperative recovery. To minimize complications, this review examines numerous preoperative strategies, including the use of antithyroid medications, beta-blockers, SSKI, and adjuvant therapy such as cholestyramine, which should be used. Patients should also be referred to a facility with a large volume of high-caliber surgical procedures. This investigation adds to the current data by comparing preoperative strategies for thyroidectomy in Graves' disease. By knowing and appropriately implementing these strategies, healthcare professionals can effectively manage Graves' disease prior to surgery, resulting in improved patient outcomes and enhanced surgical success.
